# Dietary bile acids supplementation modulates immune response, antioxidant capacity, glucose, and lipid metabolism in normal and intrauterine growth retardation piglets

**DOI:** 10.3389/fnut.2022.991812

**Published:** 2022-09-21

**Authors:** Yang Liu, Md. Abul Kalam Azad, Xiangfeng Kong, Qian Zhu, Zugong Yu

**Affiliations:** ^1^College of Veterinary Medicine, Nanjing Agricultural University, Nanjing, China; ^2^Key Laboratory of Agro-Ecological Processes in Subtropical Region, Hunan Provincial Key Laboratory of Animal Nutritional Physiology and Metabolic Process, Institute of Subtropical Agriculture, Chinese Academy of Sciences, Changsha, China

**Keywords:** antioxidant capacity, bile acid, intrauterine growth retardation, immunity, oxidative stress, weaned piglets

## Abstract

Intrauterine growth retardation (IUGR) results in intestinal dysfunction contributing to metabolic syndrome and growth lag of piglets. Bile acid (BA) presents various bioactivities, including regulation roles in antioxidant, anti-inflammation, and glucose and lipid metabolism. Forty-eight weaned piglets were allocated to four groups in a 2 × 2 factorial arrangement with the effects of BA supplementation and IUGR challenge. Twenty-four IUGR piglets and 24 normal birth weight (NBW) piglets were allocated into two groups, respectively, including the control group fed with a basal diet, and the treatment group fed a basal diet supplemented with 400 mg/kg BA. The experiment lasted 28 days. The results indicated that BA improved liver and spleen indexes in IUGR piglets, whereas decreased blood RDW-CV and RDW-SD regardless of IUGR (*P* < 0.05). Dietary BA supplementation decreased plasma CAT activity and liver GSH concentration regardless of IUGR, whereas increased plasma GSH and liver H_2_O_2_ and decreased liver T-AOC in weaned piglets (*P* < 0.05). In addition, IUGR downregulated liver *Nrf1* and *Nrf2* expression levels, while BA supplementation upregulated the *Nrf2* expression of liver in weaned piglets (*P* < 0.05). Dietary BA decreased (*P* < 0.05) jejunal GSH concentration and ileal CAT activity regardless of IUGR. Furthermore, IUGR upregulated (*P* < 0.05) jejunal *SOD* and *CAT* expression levels; however, dietary BA upregulated ileal *Nrf1* (*P* < 0.05) and *Keap1* (*P* = 0.07) expression levels in piglets regardless of IUGR. Moreover, IUGR upregulated the liver lipid synthesis (*FAS*) and downregulated *HSL* and *SCD1* expression levels, while dietary BA downregulated liver *FAS* and *SCD1* expression levels (*P* < 0.05). However, BA supplementation could enhance liver gluconeogenesis by upregulating (*P* < 0.05) the liver *G6PC* and *PCK1* expression levels in the NBW piglets but not in the IUGR piglets. Collectively, these findings suggest that BA could regulate the redox status of weaned piglets by regulating the Nrf2/Keap1 pathway and improving liver glucose and lipid metabolism of IUGR piglets. These findings will provide a reference for the application of BA in swine production; moreover, considering the physiological similarity between pigs and humans, these findings will provide a reference for IUGR research in humans.

## Introduction

Intrauterine growth retardation (IUGR) commonly results in low newborn birth weight and slow growth and development of organs (1). Previous studies reported that IUGR could lead to impaired immunity, insulin resistance, and other metabolic syndromes (2). As an important domestic economic animal, pigs exhibit higher morbidity of IUGR (15–20%), which is one of the most important reasons for enormous economic loss in swine production (3). Especially, the delay of intestinal development in IUGR piglets resulted in poor adaptability, growth performance (4), and high mortality of newborn piglets (5). A previous study showed that IUGR induced intestinal cell apoptosis and oxidative function imbalance in neonatal piglets (6). The economic loss caused by IUGR was associated with decreased neonatal immunity and immune cell counts (7). Furthermore, oxidative stress negatively affects intestinal health. Previous studies have documented that IUGR impaired the antioxidant capacity and resulted in oxidative stress in weaned piglets (8). Therefore, reducing stress and metabolic syndrome and improving the immunity and antioxidant capacity of IUGR piglets have attracted the increased attention of many researchers.

Bile acids (BAs) are cholesterol derivatives produced in the hepatocyte. Primary BAs undergo bacterial enzyme-mediated biotransformation to produce secondary BAs in the ileum and large intestine (9). BAs could regulate metabolic homeostasis, immune response, and cell proliferation *via* BA enterohepatic circulation (10). The BAs also help to modulate the gut microbiota (11), which utilizes polysaccharides and starch to generate short-chain fatty acids to meet host energy requirements. In addition, as endogenous molecules, BAs can regulate multiple physiological processes, such as energy metabolism (12), inflammatory response, and redox status (13). For example, secondary BAs can activate BA receptors, including vitamin D receptors, and modulate both the innate and adaptive immune responses (14). Previous studies showed that weaning piglets' diarrhea incidence and weight loss were prevented by activating BA receptors (15). Furthermore, BA receptors also serve as metabolic regulators and integrators of glucose, lipid, and energy metabolism (16). However, the effects of BAs on immunity and antioxidant regulation of IUGR piglets are likely less known.

The IUGR causes oxidative damage and inflammation of the intestine and then results in the abnormal metabolism of pigs (17). However, the effects of BA on the small intestine health and liver metabolism under the IUGR condition in weaning piglets remain unknown. Thus, we hypothesized that BA might improve the immune response, antioxidant ability, and glucose and lipid metabolism of weaned piglets with IUGR. Therefore, the present study was designed to determine the effects of BA on immunity, antioxidant capacity, and glucose and lipid metabolism of the small intestine and liver in weaned piglets with IUGR compared with the normal birth weight (NBW) piglets.

## Materials and methods

### Animals and experimental design

Forty-eight weaned piglets, including 24 NBW (body weight, 7.50 ± 0.29 kg) and 24 IUGR (body weight, 5.68 ± 0.43 kg) piglets from 24 L, were used in this study. Piglets with the highest birth weight (BW) and the lowest BW in the same litter were selected for the NBW and IUGR groups, respectively. After weaned at 21 d, the NBW piglets and IUGR piglets were randomly allocated to four groups (*n* = 12 per group, one pig in the IUGR+ BA group died unexpectedly): (i) NBW group, NBW piglets + basal diet; (ii) NBW+BA group, NBW piglets + basal diet with 400 mg/kg BA; (iii) IUGR group, IUGR piglets + basal diet; and (iv) IUGR+BA group, IUGR piglets + basal diet with 400 mg/kg BA. The BA (≥98.5% purity) was provided by Longchang Animal Health Care Co., Ltd. (Dezhou, China). The nutrient levels of the basal diet for all piglets met the National Research Council (18) recommendation (Supplementary Table 1). All weaned piglets were housed in a controlled temperature (23–25°C) and humidity (60 ± 5%) room and had free access to food and drinking water. Piglets were fed three times per day at 8:00, 13:00, and 18:00 with their respective diets. The experiment lasted 28 days.

### Sample collection

All piglets were weighed 12 h after the last feeding and euthanized by jugular puncture after anesthesia on d 28 of the experiment. Approximately 2 mL of blood from each pig was collected aseptically from the precaval vein into an ethylenediaminetetraacetic acid dipotassium (EDTA-K_2_) tube (Saihua, Shangdong, China) for hematological profile analysis. Blood samples (5 mL) were drawn into heparin sodium tubes (Saihua, Shangdong, China), centrifuged at 3,000 × *g* for 10 min at 4°C to obtain the plasma for cytokines and oxidant/antioxidant parameters determination. The liver and spleen were weighed, collected immediately frozen in liquid nitrogen, and transferred to ultra-low temperature refrigerator (−80°C) for total RNA extraction. The jejunum (10 cm below the flexure of duodenum-jejunum) and ileum (10 cm above the ileocecal junction) tissues were excised and rinsed with pre-cooled physiological saline for collecting the mucosa, frozen in liquid nitrogen, and immediately transferred to ultra-low temperature refrigerator (−80°C) for total RNA extraction.

### Determination of hematological profiles of blood

The hematological profiles of blood, including white blood cells (WBC), lymphocytes (LYM), intermediate cell (MID), neutrophil (NEU), lymphocyte percentage (LYM%), intermediate cell percentage (MID%), neutrophil percentage (NEU%), red blood cells (RBC), hemoglobin (HGB), hematocrit (HCT), mean corpuscular (MCV), mean corpuscular (MCH), mean corpuscular hemoglobin concentration (MCHC), red blood cell distribution width standard deviation (RDW-SD), platelets (PLT), mean platelet volume (MPV), and procalcitonin (PCT) were analyzed using an Automatic Blood Cell Analyzer (Mindray, Shenzhen, China).

### Determination of plasma cytokine and immunoglobulin concentrations

The plasma concentrations of interleukin-1 (IL-1), IL-2, IL-6, IL-10, interferon-gamma (IFN-γ), tumor necrosis factor-alpha (TNF-α), immune globulin A (IgA), (IgG), and IgM were determined using the ELISA assay kits (Huyu, Shanghai, China), according to the manufacturer's protocols. The Multiscan Spectrum Spectrophotometer (Tecan, Infinite M200 Pro, Männedorf, Switzerland) was used to detect the absorbance values.

### Determination of oxidant/antioxidant parameters of plasma, liver, and small intestinal mucosa

Approximately 0.1 g of frozen liver, ileum, and jejunum tissues were removed quickly, respectively, homogenized with ice-cold physiologic saline (1:9, w/v), and centrifuged at 2,000 × *g* for 20 min at 4°C. The supernatants were collected for oxidant/antioxidant capacity determination. The liver, ileum, jejunum, and plasma oxidant/antioxidant parameters, including catalase (CAT), glutathione (GSH), H_2_O_2_, malondialdehyde (MDA), superoxide dismutase (SOD), and total antioxidant capacity (T-AOC) were analyzed by the colorimetric method with the commercially available kits (Nanjing Jiancheng Bioengineering Institute, Nanjing, China). The oxidant/antioxidant parameters of liver, jejunum, and ileum tissues were normalized to the total protein concentration quantified by the Pierce BCA Protein Assay Kit (Beyotime Biotechnology, Shanghai, China). The Multiscan Spectrum Spectrophotometer (Tecan, Infinite M200 Pro, Männedorf, Switzerland) was used to detect the absorbance values.

### RNA isolation and real-time quantitative PCR analysis

The RNA was extracted from liver, ileum, jejunum, and spleen using TRIzol Reagent (0.1 g tissue per 1 mL TRIzol, Accurate Biology, Changsha, China). The purity and concentration of extracted total RNA were determined using a NanoDrop 2,000 spectrophotometer (Thermo Fischer Scientific, Waltham, MA, USA). The total RNA (1 μg) was reverse-transcribed using the PrimeScript RT Reagent Kit with gDNA Eraser (Accurate Biology). Then 2.0 μL of cDNA template was mixed with the forward and reverse primers (0.25 μL, respectively), SYBR Green mix (5.0 μL), and RNase free water (2.5 μL). An RT-PCR analysis was performed on the Light Cycler^®^ 480 II Real-Time PCR System (Roche, Basel, Switzerland). The real-time PCR conditions were as follows: an initial step at 95°C for 5 min, followed by 40 cycles of denaturation at 95°C for 5 s, annealing at 60°C for 30 s, and a final extension at 72°C for 30 s. Pig-specific primer sequences are shown in Supplementary Table 2 (Sangon Biotech, Shanghai, China). Target gene expression was calculated using the 2^−Δ*ΔCt*^ value (19), and β-actin was used as the internal control.

### Statistical analysis

All data were analyzed as a 2 × 2 factorial arrangement of treatments by ANOVA using the general linear model procedure of the SPSS 22.0 statistical package (SPSS Inc., Chicago, IL, USA). The statistical model included BA, IUGR, and their interactions. Differences in means in the different groups were analyzed using the Tukey-Kramer *post-hoc* test when the interaction was valid (*P* < 0.05). *P*-values < 0.05 represent significant differences. The individual piglets were considered the experimental unit. The data are expressed as means with their pooled standard error of the means (SEM).

## Results

### Effects of BA on liver and spleen indices of weaned piglets with NBW and IUGR

The effects of BA and IUGR on liver and spleen indices of weaned piglets with NBW and IUGR are shown in Table 1. The IUGR decreased (*P* < 0.05) liver weight and dietary BA increased (*P* < 0.05) liver index in weaned piglets regardless of IUGR. Dietary BA supplementation increased (*P* < 0.05) the spleen weight and index of the NBW and IUGR piglets.

**Table 1:** Effects of dietary bile acid (BA) supplementation on liver and spleen indices in weaned piglets with NBW and IUGR.

**Items**	**NBW**		**IUGR**		**SEM**	***P*** **-values**		
	**–BA**	**+BA**	**–BA**	**+BA**		**IUGR**	**BA**	IUGR*BA
Liver weight (g)	471.50	459.43	406.43	432.50	8.32	0.01	0.65	0.22
Liver index (g/kg)	25.27	27.36	26.46	29.27	0.41	< 0.01	0.04	0.62
Spleen weight (g)	32.54	37.28	30.42	35.85	1.33	0.50	0.05	0.90
Spleen index (g/kg)	1.75	2.13	2.02	2.45	0.08	0.07	0.01	0.87

Values are presented as means with their pooled SEM (*n* = 11–12). *P* < 0.05 represents significantly difference.

NBW, normal birth weight; IUGR, intrauterine growth retardation; **–**BA, a basal diet without BA; **+**BA, a basal diet supplemented with 400 mg/kg BA.

### Effects of BA on hematological profiles of weaned piglets with NBW and IUGR

The effects of BA and IUGR on hematological profiles in NBW and IUGR piglets are presented in Table 2. The number of WBC was increased (*P* < 0.05) in IUGR piglets regardless of BA. Dietary BA increased (*P* < 0.05) the PDW concentration, whereas decreased (*P* < 0.05) the percentages of blood RDW-CV and RDW-SD in the NBW and IUGR piglets.

**Table 2:** Effects of dietary bile acid (BA) supplementation on hematological profiles of weaned piglets with NBW and IUGR.

**Items**	**NBW**		**IUGR**		**SEM**	***P*** **-values**		
	**–BA**	**+BA**	**–BA**	**+BA**		**IUGR**	**BA**	IUGR*BA
WBC (10^9^/L)	13.59	12.86	16.51	14.71	4.20	0.05	0.30	0.66
HGB (g/L)	99.08	108.00	101.33	101.73	1.36	0.45	0.08	0.11
LYM (10^9^/L)	8.41	7.60	9.59	8.06	0.69	0.56	0.41	0.80
MCV (fL)	51.34	50.67	51.48	51.62	0.36	0.46	0.72	0.58
MCH (pg)	15.53	15.32	15.70	15.90	0.12	0.12	1.00	0.40
MCHC (g/L)	302.67	302.75	305.33	308.00	1.27	0.13	0.59	0.62
MON (10^9^/L)	0.39	0.50	0.43	0.54	0.05	0.73	0.73	0.98
MPV (fL)	9.03	8.96	9.11	8.93	0.11	0.91	0.58	0.80
NEU (10^9^/L)	2.29	2.67	3.05	3.61	2.39	0.24	0.51	0.90
PCT (%)	0.46	0.40	0.40	0.38	0.02	0.40	0.37	0.66
PDW (%)	15.02	15.21	14.90	15.17	0.05	0.42	0.02	0.67
PLT (10^9^/L)	509.33	447.42	439.50	426.91	24.71	0.37	0.46	0.63
RBC (10^12^/L)	6.39	7.06	6.46	6.43	0.11	0.19	0.13	0.10
RDW-CV (%)	20.82	19.83	22.18	20.21	0.34	0.18	0.03	0.45
RDW-SD (fL)	40.68	38.10	43.16	39.53	0.66	0.12	0.02	0.67

Values are expressed as means with their pooled SEM (*n* = 11–12). *P* < 0.05 represents significantly difference.

NBW, normal birth weight; IUGR, intrauterine growth retardation; –BA, a basal diet without BA; +BA, a basal diet supplemented with 400 mg/kg BA; WBC, white blood cells; LYM, lymphocytes; MID, intermediate cell; NEU, neutrophil; (LYM%), lymphocyte percentage; MID (%), intermediate cell percentage; RBC, red blood cells; HGB, hemoglobin; HCT, hematocrit; MCV, mean corpuscular; MCH, mean corpuscular; MCHC, mean corpuscular hemoglobin concentration; RDW-SD, red blood cell distribution width standard deviation; PLT, platelets; MPV, mean platelet volume; PCT, procalcitonin.

### Effects of BA on plasma cytokine and immunoglobulin indices of weaned piglets with NBW and IUGR

As presented in Table 3, dietary BA supplementation decreased (*P* < 0.05) the plasma IgM concentration in the NBW and IUGR piglets. However, there was no interaction (*P* > 0.05) of BA and IUGR in plasma cytokines and immunoglobulin concentrations of weaned piglets.

**Table 3:** Effects of dietary bile acid (BA) supplementation on plasma cytokine and immunoglobulin of weaned piglets with NBW and IUGR.

**Items**	**NBW**		**IUGR**		**SEM**	***P*** **-values**		
	**–BA**	**+BA**	**–BA**	**+BA**		**IUGR**	**BA**	IUGR*BA
IL-1 (pg/mL)	114.60	120.80	109.78	112.55	1.79	0.07	0.21	0.63
IL-2 (pg/mL)	278.50	272.38	266.45	271.68	2.02	0.12	0.91	0.16
IL-6 (pg/mL)	672.02	674.75	665.73	671.09	5.15	0.64	0.71	0.90
IL-10 (pg/mL)	129.59	129.44	125.56	132.92	2.47	0.96	0.48	0.46
INF-γ (pg/mL)	36.07	36.46	37.04	36.30	0.77	0.80	0.91	0.72
TNF-α (pg/mL)	175.49	165.15	175.51	170.31	3.93	0.29	0.81	0.29
IgA (mg/mL)	0.67	0.64	0.68	0.64	0.01	0.93	0.09	0.96
IgG (mg/mL)	19.92	21.17	20.30	20.08	0.26	0.50	0.32	0.16
IgM (mg/mL)	16.23	13.19	14.45	13.84	0.34	0.37	0.01	0.05

Values are expressed as means with their pooled SEM (*n* = 11–12). *P* < 0.05 represents significant difference.

NBW, normal birth weight; IUGR, intrauterine growth retardation; –BA, a basal diet without BA; +BA, a basal diet supplemented with 400 mg/kg BA; IL, interleukin; Ig, immunoglobulin; IFN-γ, interferon-gamma; TNF-α, tumor necrosis factor-alpha.

### Effects of BA on small intestine, spleen, and liver cytokine-related gene expression of weaned piglets with NBW and IUGR

The effects of BA and IUGR on cytokine-related gene expression in the small intestine, spleen, and liver are presented in Table 4. The IUGR downregulated (*P* < 0.05) liver *IL-6* expression level in weaned piglets, while BA supplement upregulated (*P* < 0.05) liver IL-6 expression level in the NBW piglets but not in the IUGR piglets. Dietary BA upregulated (*P* < 0.05) expression levels of spleen *IL-1*β and *IL-6*, jejunal *IL-10* and *IFN-*γ, and ileal *IL-10* and *TNF-*α of NBW and IUGR piglets. Furthermore, the liver expression levels of *IL-6* and *TNF-*α showed IUGR-BA interaction (*P* < 0.05).

**Table 4:** Effects of dietary bile acid (BA) supplementation on the intestinal and liver cytokines expressions in weaned piglets with NBW and IUGR.

**Items**	**NBW**		**IUGR**		**SEM**	***P*** **-values**		
	**–BA**	**+BA**	**–BA**	**+BA**		**IUGR**	**BA**	IUGR*BA
**Liver**								
*IL-1β*	1.00	1.12	1.12	0.90	0.11	0.91	0.89	0.51
*IL-2*	1.00	1.00	0.64	0.75	0.10	0.14	0.78	0.78
*IL-6*	1.00^ab^	1.32^a^	0.91^b^	0.71^a^	0.06	< 0.01	0.63	0.04
*IL-10*	1.00	0.86	0.66	0.86	0.07	0.22	0.82	0.21
*TNF-α*	1.00^a^	0.77^ab^	0.58^b^	0.78^ab^	0.09	0.27	0.94	< 0.05
*IFN-γ*	1.00	0.45	0.90	0.93	0.19	0.63	0.50	0.45
**Spleen**								
*IL-1β*	1.00	2.34	1.72	2.34	0.19	0.31	< 0.01	0.30
*IL-2*	1.00	1.58	1.2	1.28	0.09	0.78	0.06	0.17
*IL-6*	1.00	1.65	1.28	1.66	0.10	0.45	0.01	0.49
*IL-10*	1.00	1.27	1.26	1.43	0.09	0.26	0.23	0.79
*TNF-α*	1.00	1.17	0.99	0.93	0.05	0.22	0.60	0.24
*IFN-γ*	1.00	1.36	1.08	1.04	0.11	0.58	0.47	0.37
**Jejunum**								
*IL-1β*	1.00	1.40	1.46	1.56	0.13	0.23	0.33	0.57
*IL-2*	1.00	1.73	1.05	1.22	0.12	0.32	0.05	0.23
*IL-6*	1.00	1.12	1.02	1.08	0.06	0.92	0.50	0.82
*IL-10*	1.00	1.19	0.79	1.44	0.04	0.90	0.02	0.17
*TNF-α*	1.00	0.87	0.81	1.38	0.11	0.44	0.29	0.09
*IFN-γ*	1.00	1.44	1.17	1.60	0.10	0.40	0.03	0.97
**Ileum**								
*IL-1β*	1.00	0.97	0.88	1.22	0.09	0.16	0.14	0.50
*IL-2*	1.00	0.98	0.44	0.97	0.09	0.10	0.14	0.12
*IL-6*	1.00	1.25	1.14	1.24	0.09	0.73	0.35	0.70
*IL-10*	1.00	1.13	0.82	1.21	0.06	0.66	0.02	0.24
*TNF-α*	1.00	1.27	0.97	1.41	0.08	0.69	0.02	0.56
*IFN-γ*	1.00	1.61	1.21	1.15	1.10	0.52	0.15	0.09

Values are expressed as means with their pooled SEM (*n* = 11–12). *P* < 0.05 represents significant difference.

NBW, normal birth weight; IUGR, intrauterine growth retardation; –BA, a basal diet without BA; +BA, a basal diet supplemented with 400 mg/kg BA; *IL*, interleukin; *TNF*-α, tumor necrosis factor-α; *IFN*-γ, interferon-γ.

^a, b^Means without a common letter within the same row indicate a significant difference (*P* < 0.05).

### Effects of BA on plasma and liver oxidant/antioxidant indices of weaned piglets with NBW and IUGR

The effects of IUGR and BA on plasma and liver oxidant/antioxidant indices of weaned piglets with NBW and IUGR are shown in Table 5. Dietary BA decreased (*P* < 0.05) plasma CAT activity and liver GSH concentration regardless of IUGR. Dietary BA decreased (*P* < 0.05) liver T-AOC concentration, whereas increased (*P* < 0.05) the plasma GSH and liver H_2_O_2_ concentrations in weaned piglets regardless of IUGR. Furthermore, there were interactions (*P* < 0.05) between BA and IUGR in the plasma T-AOC concentration and liver CAT activity in weaned piglets.

**Table 5:** Effects of dietary bile acid (BA) supplementation on plasma and liver oxidant/antioxidant indices in weaned piglets with NBW and IUGR.

**Items**	**NBW**		**IUGR**		**SEM**	***P*** **-values**		
	**–BA**	**+BA**	**–BA**	**+BA**		**IUGR**	**BA**	IUGR*BA
**Plasma**								
CAT (U/mL)	9.96	8.90	11.02	8.75	0.33	0.47	0.01	0.33
SOD (U/mL)	12.82	13.20	12.79	12.87	0.18	0.63	0.54	0.07
H_2_O_2_ (mmol/L)	25.45	27.91	29.11	31.09	1.84	0.37	0.56	0.95
GSH (μmol/L)	12.35	14.95	16.33	18.55	0.45	< 0.01	0.01	0.83
MDA (nmol/mL)	7.99	11.66	9.71	11.02	0.62	0.77	0.28	0.87
T-AOC (mmol/mL)	0.19^a^	0.11^b^	0.10^b^	0.18^ac^	0.01	0.67	0.87	< 0.01
**Liver**								
CAT (U/mgprot)	60.88^ab^	61.13^ab^	63.11^a^	53.12^b^	1.90	0.45	0.20	0.02
GSH (μmol/mgprot)	1.09	0.73	1.07	0.74	0.04	0.85	< 0.01	0.81
H_2_O_2_ (mmol/gprot)	55.24	70.34	63.40	74.00	2.73	0.27	0.02	0.67
MDA (nmol/mgprot)	0.20	0.20	0.23	0.24	0.01	0.08	0.65	0.74
SOD (U/mgprot)	29.21	28.86	31.22	28.59	0.61	0.48	0.23	0.35
T-AOC (mmol/gprot)	0.60	0.43	0.56	0.46	0.02	0.99	< 0.01	0.44

Values are expressed as means with their pooled SEM (*n* = 11–12). *P* < 0.05 represents significant difference.

NBW, normal birth weight; IUGR, intrauterine growth retardation; –BA, a basal diet without BA; +BA, a basal diet supplemented with 400 mg/kg BA; CAT, catalase; GPX, glutathione peroxidase; MDA, malondialdehyde; SOD, superoxide dismutase; T-AOC, total antioxidant capacity.

^a, b, c^Means without a common letter within the same row indicate a significant difference (*P* < 0.05).

### Effects of BA on liver antioxidant-related gene expression of weaned piglets with NBW and IUGR

The effects of IUGR and BA on liver antioxidant-related gene expression in weaned piglets with NBW and IUGR are shown in Table 6. The IUGR downregulated (*P* < 0.05) the expression levels of liver *Nrf1* and *Nrf2* in weaned piglets, while dietary BA supplementation upregulated (*P* < 0.05) only the expression level of liver *Nrf2* in weaned piglets. However, there were no interactions (*P* > 0.05) between BA and IUGR on liver antioxidant-related gene expressions in weaned piglets.

**Table 6:** Effects of dietary bile acid (BA) supplementation on liver antioxidant-related gene expression in weaned piglets with NBW and IUGR.

**Items**	**NBW**		**IUGR**		**SEM**	***P*** **-values**		
	**–BA**	**+BA**	**–BA**	**+BA**		**IUGR**	**BA**	IUGR*BA
*CAT*	1.00	0.95	0.83	0.81	0.06	0.17	0.71	0.91
*GPX*	1.00	1.20	0.92	1.02	0.07	0.38	0.31	0.74
*SOD*	1.00	1.49	1.16	1.22	0.07	0.73	0.08	0.16
*Nrf1*	1.00	1.10	0.80	0.82	0.05	0.03	0.55	0.69
*Nrf2*	1.00	1.84	0.46	1.35	0.13	0.04	< 0.01	0.93
*Keap1*	1.00	1.23	0.99	1.16	0.07	0.46	0.07	0.52

Values are expressed as means with their pooled SEM (*n* = 11–12). *P* < 0.05 represents significant difference.

NBW, normal birth weight; IUGR, intrauterine growth retardation; –BA, a basal diet without BA; +BA, a basal diet supplemented with 400 mg/kg BA; *CAT*, catalase; *GPX*, glutathione peroxidase; *Keap1*, kelch-like ECH-associated protein 1; *Nrf1*, nuclear factor erythroid 2-related factor 1; *Nrf2*, nuclear factor erythroid 2-related factor; SOD, superoxide dismutase.

### Effects of BA on jejunal and ileal oxidant/antioxidant indices of weaned piglets with NBW and IUGR

The effects of BA and IUGR on jejunal and ileal oxidant/antioxidant indices of weaned piglets with NBW and IUGR are shown in Table 7. Dietary BA supplementation decreased (*P* < 0.05) GSH concentration in the jejunum and ileum. Furthermore, dietary BA supplementation to weaned piglets decreased (*P* < 0.05) the ileal CAT activity. There was interaction (*P* < 0.05) between BA and IUGR in the ileal GSH concentration in weaned piglets.

**Table 7:** Effects of dietary bile acid (BA) supplementation on the small intestine oxidant/antioxidant indices in weaned piglets with NBW and IUGR.

**Items**	**NBW**		**IUGR**		**SEM**	***P*** **-values**		
	**–BA**	**+BA**	**–BA**	**+BA**		**IUGR**	**BA**	IUGR*BA
**Jejunum**								
CAT (U/mgprot)	5.53	5.54	6.02	4.88	0.2	0.84	0.17	0.13
GSH (μmol//gprot)	89.77	73.47	93.26	68.58	2.44	0.85	< 0.01	0.26
MDA (nmol//mgprot)	1.39	1.27	1.24	1.08	0.05	0.11	0.17	0.89
SOD (U/mgprot)	3.06	3.13	3.67	3.10	0.09	0.08	0.12	0.05
**Ileum**								
CAT (U/mgprot)	3.09	2.22	3.06	2.71	0.11	0.27	< 0.01	0.20
GSH (μmol//gprot)	74.85^a^	68.04^b^	83.06^ac^	63.04^b^	1.77	0.56	< 0.01	0.02
MDA (nmol//mgprot)	1.22	1.06	1.23	1.18	0.03	0.33	0.13	0.44
SOD (U/mgprot)	3.37	3.86	3.66	3.57	0.08	0.99	0.17	0.06

Values are expressed as means with their pooled SEM (*n* = 11–12). *P* < 0.05 represents significant difference.

NBW, normal birth weight; IUGR, intrauterine growth retardation; –BA, a basal diet without BA; +BA, a basal diet supplemented with 400 mg/kg BA; CAT, catalase; GSH, glutathione; MDA, malondialdehyde; SOD, superoxide dismutase.

^a, b, c^Means without a common letter within the same row indicate a significant difference (*P* < 0.05).

### Effects of BA on small intestinal antioxidant-related gene expression of weaned piglets with NBW and IUGR

As shown in Table 8, IUGR upregulated (*P* < 0.05) the jejunal *CAT* expression level, while dietary BA supplementation to IUGR piglets downregulated (*P* < 0.05) the jejunal *CAT* expression level. Dietary BA supplementation to IUGR piglets upregulated (*P* < 0.05) the jejunal SOD expression level, while BA supplementation to NBW piglets upregulated (*P* < 0.05) the jejunal *GPX* expression level. Moreover, dietary BA supplementation upregulated (*P* < 0.05) the ileal *Nrf1* and *Nrf2* expression levels regardless of IUGR. There were interactions (*P* < 0.05) between BA and IUGR on the ileal *GPX* and *Nrf2* expression levels and jejunal *SOD* and *GPX* expression levels in weaned piglets.

**Table 8:** Effects of dietary bile acid (BA) supplementation on the small intestinal antioxidant-related gene expressions in weaned piglets with NBW and IUGR.

**Items**	**NBW**		**IUGR**		**SEM**	***P*** **-values**		
	**–BA**	**+BA**	**–BA**	**+BA**		**IUGR**	**BA**	IUGR*BA
**Jejunum**								
*SOD*	1.00^c^	1.45^ab^	1.22^abc^	1.48^a^	0.08	0.02	0.11	0.01
*GPX*	1.00^b^	1.42^a^	1.07^b^	1.37^ab^	0.08	< 0.01	0.10	< 0.01
*CAT*	1.00	1.38	1.86	1.17	0.10	0.07	0.02	0.16
*Nrf1*	1.00	1.09	0.92	0.99	0.04	0.28	0.37	0.90
*Nrf2*	1.00	1.22	1.03	1.12	0.06	0.75	0.21	0.60
*Keap1*	1.00	1.02	0.99	1.06	0.05	0.85	0.68	0.85
**Ileum**								
*CAT*	1.00	1.23	1.08	1.07	0.05	0.97	0.12	0.12
*GPX*	1.00^b^	1.39^a^	1.00^b^	1.17^ab^	0.08	0.47	0.07	0.01
*SOD*	1.00	0.90	0.80	0.84	0.05	0.17	0.72	0.47
*Nrf1*	1.00	1.39	0.99	1.34	0.06	0.27	0.03	0.32
*Nrf2*	1.00^b^	1.59^a^	1.18^b^	1.06^b^	0.08	0.22	0.09	0.02
*Keap1*	1.00	1.60	1.02	1.29	0.07	0.25	< 0.01	0.19

Values are expressed as means with their pooled SEM (*n* = 11–12). *P* < 0.05 represents significant difference.

NBW, normal birth weight; IUGR, intrauterine growth retardation; –BA, a basal diet without BA; +BA, a basal diet supplemented with 400 mg/kg BA; *CAT*, catalase; *GPX*, glutathione peroxidase; *Keap1*, kelch-like ECH-associated protein 1; *Nrf1*, nuclear factor erythroid 2-related factor 1; *Nrf2*, nuclear factor erythroid 2-related factor; *SOD*, superoxide dismutase.

^a, b, c^Means without a common letter within the same row indicate a significant difference (*P* < 0.05).

### Effects of BA on liver bile acid, glucose, and lipid metabolism-related gene expression of weaned piglets with NBW and IUGR

As shown in Table 9, IUGR upregulated (*P* < 0.05) liver fatty acid synthase (*FAS*) expression level and downregulated (*P* < 0.05) liver pyruvate carboxylase (*PC*) and hormone-sensitive lipase (*HSL*) expression levels in weaned piglets. Dietary BA supplementation upregulated (*P* < 0.05) liver glucose-6-phosphatase (*G6PC*), *PC*, and phosphoenolpyruvate carboxykinase 1 (*PCK1*) expression levels, whereas downregulated (*P* < 0.05) liver cytochrome P450 family 8 subfamily A member 1 (*CYP7A1*), *FAS*, and stearoyl-coenzyme A desaturase 1 (SCD1) expression levels regardless of IUGR. Furthermore, there was interaction (*P* < 0.05) between BA and IUGR on the liver *PC* expression level in weaned piglets.

**Table 9:** Effects of dietary bile acid (BA) supplementation on liver BA, glucose, and lipid metabolism-related gene expressions in weaned piglets with NBW and IUGR.

**Items**	**NBW**		**IUGR**		**SEM**	***P*** **-values**		
	**–BA**	**+BA**	**–BA**	**+BA**		**IUGR**	**BA**	IUGR*BA
**Genes related to BA metabolism**								
*CYP7A1*	1.00	0.27	1.20	0.78	0.14	0.20	0.04	0.57
*CYP8B1*	1.00	0.81	1.51	1.63	0.22	0.16	0.94	0.74
*CYP27A1*	1.00	0.85	0.85	0.79	0.06	0.34	0.36	0.69
*FXR*	1.00	1.09	0.73	0.82	0.07	0.06	0.52	0.99
*NTCP*	1.00	0.80	0.86	0.69	0.08	0.44	0.24	0.93
**Genes related to gluconeogenesis**								
*G6PC*	1.00	2.75	1.38	1.55	0.22	0.36	0.04	0.08
*PC*	1.00^b^	3.20^a^	1.28^b^	1.44^b^	0.20	< 0.05	< 0.01	< 0.01
*PCK1*	1.00	2.59	1.26	2.17	0.24	0.86	< 0.01	0.45
*PCK2*	1.00	1.55	1.85	1.29	0.15	0.35	0.99	0.09
**Genes related to lipid metabolism**								
*ACC*	1.00	2.05	1.63	1.16	0.20	0.75	0.47	0.07
*ATGL*	1.00	1.36	1.19	1.26	0.11	0.82	0.32	0.51
*FAS*	1.00	0.52	1.37	0.94	0.10	0.04	0.02	0.88
*HSL*	1.00	1.48	0.66	0.81	0.10	0.02	0.12	0.40
*SCD1*	1.00	0.66	0.89	0.56	0.07	0.44	< 0.05	0.99
*SREBP/c*	1.00	0.45	0.89	0.86	0.10	0.43	0.14	0.17

Values are expressed as means with their pooled SEM (*n* = 11–12). *P* < 0.05 represents significant difference.

NBW, normal birth weight; IUGR, intrauterine growth retardation; –BA, a basal diet without BA; +BA, a basal diet supplemented with 400 mg/kg *BA*; ACC, acetyl-CoA carboxylase; *ATGL*, adipose triglyceride lipase; *BESP*, bile salt export pump; *CAT*, catalase; *CYP7A1*, cytochrome P450 family 7 subfamily A member 1; *CYP8B1*, cytochrome P450 family 8 subfamily B member 1; *CYP27A1*, cytochrome P450 family 27 subfamily A member 1; *FAS*, fatty acid synthase; *FXR*, farnesoid X-activated receptor; *GPX*, glutathione peroxidase; *G6PC*, glucose-6-phosphatase; *HSL*, hormone-sensitive lipase; *NTCP*, sodium taurocholate cotransporting polypeptide; *PC*, pyruvate carboxylase; *PCK1*, phosphoenolpyruvate carboxykinase 1; *PCK2*, phosphoenolpyruvate carboxykinase 2; *SCD1*, stearoyl-Coenzyme A desaturase 1; *SREBP/c*, sterol regulatory element binding protein c.

^a, b^Means without a common letter within the same row indicate a significant difference (*P* < 0.05).

## Discussion

The IUGR is a major concern in swine production, which is associated with the increased mortality of newborn piglets and has permanent adverse effects on postnatal growth and the long-term health of pigs. Knowledge of the underlying mechanisms contributed to prevent IUGR and improve animal health and swine production efficiency. The BAs are synthesized in hepatocytes and are an important regulator that connects the liver and intestine. The enterohepatic circulation of BAs participates in regulating the individual hepatic and intestinal functions (10). This study aimed to explore the effects of BA on immunity response, redox status, and glucose and lipid metabolism in weaned piglets with IUGR. In the present study, supplementation of BA to NBW and IUGR piglets had no significant impact on growth performance. However, we found that BA supplementation had beneficial effects on the immune regulatory and antioxidant capacity in NBW piglets, whereas dietary BA supplementation to IUGR piglets decreased lipid synthesis and enhanced metabolic syndrome in weaned piglets.

The IUGR caused by maternal undernutrition or placental insufficiency is mainly associated with reduced organs and tissues (20). Proteomics analysis revealed that the high level of hepatic proteins was associated with the increased intestinal digestion and absorption of nutrients, as well as oxidative stress and immune response (21). The liver participates various physiological processes, such as immune response and lipid and cholesterol metabolism. Furthermore, the liver oxidizes lipids and binds excess lipid storage in adipose tissues (22). In the present study, IUGR decreased the liver weight and increased the liver index of weaned piglets, which was in accordance with a previous study (2). In addition, dietary BA to IUGR piglets increased the liver index. These findings show that BA could improve liver development and function of weaned piglets with IUGR. The spleen has important functions in immunity and hematopoiesis (23). Consistent with a previous study, IUGR reduced the spleen weight of weaned piglets in the present study (24). Moreover, dietary BA supplementation increased the spleen index in both NBW and IUGR piglets. These findings indicate that BA could improve spleen development and function to some extent. However, more research are needed to investigate the exact mechanism.

The hematological profiles are often used to evaluate animal health. The number of WBC can indicate systemic inflammation, and a high level of WBC indicates that inflammation was occurring (25). In the present study, IUGR increased the number of WBC in weaned piglets; dietary BA supplementation decreased the number of WBC whereas increased the percentages of blood PDW in IUGR piglets. The results indicate that BA might improve the immune response of IUGR piglets. Inflammation is an important indicator of animal health. However, there were no significant changes in the plasma, jejunal, and ileal cytokine concentrations in the present study. Collectively, these findings showed that BA seems to have the effect of improving the ability of bone marrow by producing more blood and thus could improve the immune response of IUGR piglets. However, further investigation would be more worth to confirm this.

The plasma antioxidant capacity indicated the host's systemic ability to resist oxidative damage (18). H_2_O_2_ is an important type of reactive oxygen species and is associated with lipoperoxidation (26). The MDA also is one of the final products of lipoperoxidation and is an important marker of oxidative stress (27). The GSH-dependent enzyme included the SOD, GSH-Px, and CAT, which participate in scavenging reactive oxygen species. IUGR can decrease the plasma T-AOC concentration of piglets, while dietary BA supplementation to IUGR piglets increased the plasma T-AOC concentration in this study. Furthermore, dietary BA increased the plasma CAT activity in the NBW piglets and the GSH concentration in the NBW and IUGR piglets. These results suggest that dietary BA had potential antioxidant effects on IUGR piglets.

The IUGR can also cause intestinal oxidative stress. A previous study showed that IUGR can increase the ileal MDA and H_2_O_2_ concentrations, whereas decreased jejunal CAT activity, T-AOC, and GSH concentrations, and ileal CAT activity in piglets (27). Our study showed that IUGR increased the jejunal SOD activity and ileal GSH concentration, which is in accordance with a previous study (28). Dietary BA to IUGR piglets decreased the jejunal GSH, SOD, activities and ileal GSH concentration; however, there are no significant differences in the gene expression related to the small intestinal antioxidant ability of piglets. Furthermore, our results also indicated that dietary BA increased small intestine antioxidant capacity in the NBW piglets and upregulated expression levels of antioxidant-related genes, including jejunal *SOD, GPX*, and ileal *GPX, Nrf2*, and *Keap1*. Collectively, dietary BA improved the jejunal and ileal antioxidant ability of NBW piglets, which was associated with Nrf1/2 pathways. However, those gene expression levels were decreased in the IUGR piglets. Considering the poor physical condition of IUGR piglets, the selection of appropriate BA doses may help to improve the antioxidant capacity of IUGR piglets. These findings suggest that BA could reduce intestinal oxidative damage caused by IUGR and improve antioxidant ability by Nrf1/2 signaling pathways.

The BAs were used as metabolic regulators *via* their receptors to regulate hepatic lipid and glucose homeostasis (29). The CYP7A1 catalyzes BA synthesis in the liver (30). The nuclear farnesoid X receptor (FXR) has negative feedback to inhibit the *CYP7A1* expression and BA synthesis (31). We found that IUGR could upregulate hepatic *CYP7A1* expression level, while dietary BA supplementation to IUGR piglets downregulated hepatic *CYP7A1* expression level. However, other pathways related to BA synthesis did not significantly change in the present study. These findings suggest that dietary BA could reduce hepatic BA synthesis in IUGR piglets by inhibiting the classical pathway of BA synthesis to prevent BA accumulation in the liver.

Previous studies showed that IUGR increased serum insulin level and lipid accumulation in the liver, which were associated with the increased hepatic fatty acid synthase (32). A previous study suggested that BAs participate in glucose metabolism *via* regulating the expression of gluconeogenesis-related genes (33). In the present study, dietary BA upregulated hepatic *G6PC* and *PCK1* expression levels, which was related to gluconeogenesis in weaned piglets regardless of IUGR, while BA supplementation upregulated the expression level of hepatic *PC* in the NBW piglets. These findings indicate that BA could enhance liver gluconeogenesis in the NBW piglets but not in the IUGR piglets.

Piglets with IUGR generally suffer from abnormal lipid metabolism and insulin resistance (32). Emerging evidence showed that dietary BA could regulate the expression of some key genes related to lipid metabolisms (34), including *ACC* and *FAS* (35). We found that IUGR upregulated hepatic gene expression levels related to *FAS*, while downregulated the genes related to lipid degradation (including *HSL* and *SCD1*). These results indicate that a higher lipid level in the IUGR pigs was associated with increased lipid synthesis. Furthermore, dietary BA supplementation to IUGR piglets downregulated hepatic lipid synthesis gene expression levels, indicating that IUGR could increase lipid synthesis, while dietary BA might have the ability to inhibit hepatic lipid accumulation. Therefore, as a potential regulator, dietary BA can improve the disorder of lipid metabolism and metabolic syndrome of IUGR piglets.

## Conclusion

In summary, dietary BA had beneficial effects on immune regulatory and antioxidant capacity in NBW piglets, whereas dietary BA supplementation to IUGR piglets decreased lipid synthesis and enhanced metabolic syndrome. These findings provide new insights that BA supplementation is a promising strategy for improving the immunity and antioxidant capacity of weaned piglets. Moreover, considering the physiological similarity between pigs and humans, our findings will provide new insight into the prevention and treatment of IUGR in animals.

## Data availability statement

The original contributions presented in the study are included in the article/Supplementary materials, and further inquiries can be directed to the corresponding authors.

## Ethics statement

The animal study was reviewed and approved by the Animal Care and Use Committee of the Institute of Subtropical Agriculture, Chinese Academy of Sciences.

## Author contributions

XK and ZY designed the experiments. YL and QZ conducted the experiments. YL and XK wrote the manuscript. XK, MA, ZY, and YL revised the manuscript. All authors read and approved the final manuscript.

## Funding

This study was jointly supported by the National Natural Science Foundation of China (U20A2056), China Agriculture Research System of MOF and MARA (CARS-35), and Special Funds for Construction of Innovative Provinces in Hunan Province (2019RS3022).

## Conflict of interest

The authors declare that the research was conducted in the absence of any commercial or financial relationships that could be construed as a potential conflict of interest.

## Publisher's note

All claims expressed in this article are solely those of the authors and do not necessarily represent those of their affiliated organizations, or those of the publisher, the editors and the reviewers. Any product that may be evaluated in this article, or claim that may be made by its manufacturer, is not guaranteed or endorsed by the publisher.

## References

[1] Wu G Bazer FW Wallace JM Spencer TE. Board-invited review: Intrauterine growth retardation: Implications for the animal sciences. J Anim Sci. (2006) 84:2316. 10.2527/jas.2006-156 16908634

[2] Niu Y He J Zhao Y Shen M Zhang L Zhong X. Effect of curcumin on growth performance, inflammation, insulin level, and lipid metabolism in weaned piglets with IUGR. Animals. (2019) 9:1098. 10.3390/ani9121098 31818040 PMC6940831

[3] Dong L Zhong X He J Zhang L Bai K Xu W. Supplementation of tributyrin improves the growth and intestinal digestive and barrier functions in intrauterine growth-restricted piglets. Clin Nutr. (2016) 35:399. 10.1016/j.clnu.2015.03.002 26112894

[4] Wang J Chen L Li D Yin Y Wang X Li P. Intrauterine growth restriction affects the proteomes of the small intestine, liver, and skeletal muscle in newborn pigs. J Nutr. (2008) 138:60. 10.1093/jn/138.1.60 18156405

[5] Che L Zhou Q Liu Y Hu L Peng X Wu C. Flaxseed oil supplementation improves intestinal function and immunity, associated with altered intestinal microbiome and fatty acid profile in pigs with intrauterine growth retardation. Food Funct. (2019) 10:8149. 10.1039/C9FO01877H 31696186

[6] Tang X Xiong K. Intrauterine growth retardation affects intestinal health of suckling piglets *via* altering intestinal antioxidant capacity, glucose uptake, tight junction, and immune responses. Oxid Med Cell Longev. (2022) 2022:2644205. 10.1155/2022/2644205 35345830 PMC8957421

[7] Zarate MA De Dios RK Balasubramaniyan D Zheng L Sherlock LG Rozance PJ. The acute hepatic NF-κB-mediated proinflammatory response to endotoxemia is attenuated in intrauterine growth-restricted newborn mice. Front Immunol. (2021) 12:706774. 10.3389/fimmu.2021.706774 34539638 PMC8440955

[8] Zhang L Zhang J Yan E He J Zhong X Zhang L. Dietary supplemented curcumin improves meat quality and antioxidant status of intrauterine growth retardation growing pigs *via* Nrf2 signal pathway. Animals. (2020) 10:539. 10.3390/ani10030539 32213933 PMC7143559

[9] Ridlon JM Kang DJ Hylemon PB. Bile salt biotransformations by human intestinal bacteria. J Lipid Res. (2006) 47:241. 10.1194/jlr.R500013-JLR200 16299351

[10] Li T Chiang JY. Bile acid signaling in metabolic disease and drug therapy. Pharmacol Rev. (2014) 66:948. 10.1124/pr.113.008201 25073467 PMC4180336

[11] Topping DL Clifton PM. Short-chain fatty acids and human colonic function: roles of resistant starch and nonstarch polysaccharides. Physiol Rev. (2001) 81:1031. 10.1152/physrev.2001.81.3.1031 11427691

[12] Reschly EJ Ai N Ekins S Welsh WJ Hagey LR Hofmann AF. Evolution of the bile salt nuclear receptor FXR in vertebrates. J Lipid Res. (2008) 49:1577. 10.1194/jlr.M800138-JLR200 18362391 PMC2431107

[13] Wu H Liu G He Y Da J Xie B. Obeticholic acid protects against diabetic cardiomyopathy by activation of FXR/Nrf2 signaling in db/db mice. Eur J Pharmacol. (2019) 858:172393. 10.1016/j.ejphar.2019.05.022 31085240

[14] Aranow C. Vitamin D and the immune system. J Investig Med. (2011) 59:881. 10.2310/JIM.0b013e31821b8755 21527855 PMC3166406

[15] Cantorna MT Munsick C Bemiss C Mahon BD. 1,25-Dihydroxycholecalciferol prevents and ameliorates symptoms of experimental murine inflammatory bowel disease. J Nutr. (2000) 130:2648. 10.1093/jn/130.11.2648 11053501

[16] Chiang JYL Ferrell JM. Discovery of farnesoid X receptor and its role in bile acid metabolism. Mol Cell Endocrinol. (2022) 548:111618. 10.1016/j.mce.2022.111618 35283218 PMC9038687

[17] Zhang H Li Y Wang T. Antioxidant capacity and concentration of redox-active trace mineral in fully weaned intra-uterine growth retardation piglets. J Anim Sci Biotechnol. (2015) 6:48. 10.1186/s40104-015-0047-7 26587234 PMC4652383

[18] National Reaserch Couuncil. Nutrient Requirements of Swine. 11th revised. Washington, DC: National Academies Press (2012).

[19] Wang K Kong X Azad MAK Zhu Q Xiong L Zheng Y. Maternal probiotic or synbiotic supplementation modulates jejunal and colonic antioxidant capacity, mitochondrial function, and microbial abundance in Bama mini-piglets. Oxid Med Cell Longev. (2021) 2021:6618874. 10.1155/2021/6618874 34035877 PMC8116152

[20] Greenwood PL Bell AW. Consequences of intra-uterine growth retardation for postnatal growth, metabolism and pathophysiology. Reprod Suppl. (2003) 61:195. 10.1530/biosciprocs.5.015 14635936

[21] Wang X Wu W Lin G Li D Wu G Wang J. Temporal proteomic analysis reveals continuous impairment of intestinal development in neonatal piglets with intrauterine growth restriction. J Proteome Res. (2010) 9:924. 10.1021/pr900747d 19938879

[22] Trefts E Gannon M Wasserman DH. The liver. Curr Biol. (2017) 27:R1147. 10.1016/j.cub.2017.09.019 29112863 PMC5897118

[23] Brendolan A Rosado MM Carsetti R Selleri L Dear TN. Development and function of the mammalian spleen. Bioessays. (2007) 29:166. 10.1002/bies.20528 17226804

[24] Zhang W Ma C Xie P Zhu Q Wang X Yin Y. Gut microbiota of newborn piglets with intrauterine growth restriction have lower diversity and different taxonomic abundances. J Appl Microbiol. (2019) 127:354. 10.1111/jam.14304 31077497 PMC6916403

[25] Lewis SM Williams A Eisenbarth SC. Structure and function of the immune system in the spleen. Sci Immunol. (2019) 4:eaau6085. 10.1126/sciimmunol.aau6085 30824527 PMC6495537

[26] Mittler R Vanderauwera S Suzuki N Miller G Tognetti VB Vandepoele K. ROS signaling: the new wave? Trends Plant Sci. (2011) 16:300. 10.1016/j.tplants.2011.03.007 21482172

[27] Pirinccioglu AG Gökalp D Pirinccioglu M Kizil G Kizil M. Malondialdehyde (MDA) and protein carbonyl (PCO) levels as biomarkers of oxidative stress in subjects with familial hypercholesterolemia. Clin Biochem. (2010) 43:1220. 10.1016/j.clinbiochem.2010.07.022 20691171

[28] Yan E Zhang J Han H Wu J Gan Z Wei C. Curcumin alleviates IUGR jejunum damage by increasing antioxidant capacity through Nrf2/Keap1 pathway in growing pigs. Animals. (2019) 10:41. 10.3390/ani10010041 31878265 PMC7022777

[29] Chiang JY. Bile acid metabolism and signaling. Compr Physiol. (2013) 3:1191. 10.1002/cphy.c120023 23897684 PMC4422175

[30] Pandak WM Schwarz C Hylemon PB Mallonee D Valerie K Heuman DM. Effects of *CYP7A1* overexpression on cholesterol and bile acid homeostasis. Am J Physiol Gastrointest Liver Physiol. (2001) 281:G878. 10.1152/ajpgi.2001.281.4.G878 11557507

[31] Chiang JYL Ferrell JM. Bile acid receptors FXR and TGR5 signaling in fatty liver diseases and therapy. Am J Physiol Gastrointest Liver Physiol. (2020) 318:G554. 10.1152/ajpgi.00223.2019 31984784 PMC7099488

[32] He J Dong L Xu W Bai K Lu C Wu Y. Dietary tributyrin supplementation attenuates insulin resistance and abnormal lipid metabolism in suckling piglets with intrauterine growth retardation. PLoS ONE. (2015) 10:e0136848. 10.1371/journal.pone.0136848 26317832 PMC4552672

[33] Shapiro H Kolodziejczyk AA Halstuch D Elinav E. Bile acids in glucose metabolism in health and disease. J Exp Med. (2018) 215:383. 10.1084/jem.20171965 29339445 PMC5789421

[34] Jiang T Wang XX Scherzer P Wilson P Tallman J Takahashi H. Farnesoid X receptor modulates renal lipid metabolism, fibrosis, and diabetic nephropathy. Diabetes. (2007) 56:2485. 10.2337/db06-1642 17660268

[35] Chen J Zhou Y Zhuang Y Qin T Guo M Jiang J. The metabolic regulator small heterodimer partner contributes to the glucose and lipid homeostasis abnormalities induced by hepatitis C virus infection. Metabolism. (2019) 100:153954. 10.1016/j.metabol.2019.153954 31400386

