# Supplementary material for: Dietary bile acids supplementation modulates immune response, antioxidant capacity, glucose, and lipid metabolism in normal and intrauterine growth retardation piglets
Source: Front Nutr. 2022 Sep 21;9:991812. doi: 10.3389/fnut.2022.991812 (PMC9534482; doi:10.3389/fnut.2022.991812)
Supplement: Supplementary file 1 [file Data_Sheet_1.docx]

**Supplementary Table 1.** Ingredients and nutrient levels of a basal diet (%, as-fed basis)

| Items | Content |
| --- | --- |
| Ingredients | |
| Corn | 48.5 |
| Extruded soybean | 12.00 |
| Extruded corn | 10.00 |
| Soybean meal | 7.50 |
| Fermented soybean meal | 5.00 |
| Whey powder | 5.00 |
| Steam fish meal | 3.00 |
| Oil powder | 2.00 |
| Glucose | 2.00 |
| Premix ^1^ | 5.00 |
| Total | 100.00 |
| Nutrient levels ^2^ | |
| Digestible energy (MJ/kg) | 14.44 |
| Metabolizable energy (MJ/kg) | 13.81 |
| Crude protein | 17.50 |
| Crude fat | 4.70 |
| Ash | 4.10 |
| Crude fiber | 2.34 |
| Digestible sulfur-containing amino acids/lysine | 0.55 |
| Digestible threonine/lysine | 0.65 |
| Digestible tryptophan/lysine | 0.19 |

^1^ The premix provided the following per kilogram complete diet: vitamin A 12,000 IU; vitamin D_3_ 3,000 IU; vitamin E 50 mg; vitamin K_3_ 4 mg; vitamin B_1_ 4 mg; vitamin B_2_ 10 mg; vitamin B_6_ 7 mg; vitamin B_12_ 0.05 mg; acidifier 5.00 g; choline chloride 1.00 g; Cu (CuSO_4_ 5H_2_O) 0.50 g; Mn (MnSO_4_ H_2_O) 0.30 g; Zn (ZnSO_4_ H_2_O) 0.30 g; Fe (FeSO_4_ H_2_O) 0.60 g; I (KIO_3_) 10 mg; Se (Na_2_SeO_3_) 1% 10 mg.

^2^ Nutrient levels were calculated values.

**Supplementary Table** **2.** Pig-specific primer sequences used for RT-PCR

| Genes |  | Primer sequences (5′ to 3′) | Product size (bp) | Accession No. |
| --- | --- | --- | --- | --- |
| *ACC* | Forward | GGCCATCAAGGACTTCAACC | 102 | NM_001114269.1 |
|  | Reverse | ACGATGTAAGCGCCGAACTT |  |  |
| *ATGL* | Forward | TGTTCCCCAAAGAGACGACG | 117 | NM_001098605.1 |
|  | Reverse | CGTTGGCCACTAGGGAGGA |  |  |
| *CAT* | Forward | AGCCTACGTCCTGAGTCTCTGC | 90 | NM_214301.2 |
|  | Reverse | TCCATATCCGTTCATGTGCCTGTG |  |  |
| *CYP27A1* | Forward | TTGAGAAACGCATTGGCTGC | 155 | NM_001243304 |
|  | Reverse | ATCCAGGTATCGCCTCCAGT |  |  |
| *CYP7A1* | Forward | TCCGGAATCTACACGACAGC | 192 | NM_001005352.3 |
|  | Reverse | CCACAGGTATCAGAAGCGGAT |  |  |
| *CYP8B1* | Forward | GCAGGCGGAGGAGTTATTCA | 120 | NM_214426.1 |
|  | Reverse | TTATGCCGTGCCTCTCCAAG |  |  |
| *FXR* | Forward | TGGGAATGTTGGCTGAATGT | 92 | NM_001287412 |
|  | Reverse | GATCTGCATGCTGCTTCACG |  |  |
| *G6PC* | Forward | GTGATCGCGGACCTCAGAAA | 110 | NM_001113445.1 |
|  | Reverse | AGCCAGTCTCCAATCACAGC |  |  |
| *GPX* | Forward | TGAATGGCGCAAATGCTCAC | 161 | NM_001043534.1 |
|  | Reverse | ATTGCGACACACTGGAGACC |  |  |
| *HSL* | Forward | ACCCTCGGCTGTCAACTTCTT | 157 | NM_214315.3 |
|  | Reverse | TCCTCCTTGGTGCTAATCTCGT |  |  |
| *IFN-γ* | Forward | GGCCATTCAAAGGAGCATGGA | 118 | NM_213948.1 |
|  | Reverse | TCACTGATGGCTTTGCGCT |  |  |
| *IL-1β* | Forward | CCGCCAAGATATAACTGAC | 124 | NM_214055.1 |
|  | Reverse | GCAGCAACCATGTACCAA |  |  |
| *IL-2* | Forward | AGCTCTGGAGGGAGTGCTAA | 156 | NM_213861.1 |
|  | Reverse | ACAGCAGTTACTGTCTCATCATCA |  |  |
| *IL-6* | Forward | GGATTTCCTGCAGTTCAGCCT | 102 | NM_214399.1 |
|  | Reverse | ACAGGTTTCTGACCAGAGGAG |  |  |
| *IL-10* | Forward | ATGGGCGACTTGTTGCTGAC | 154 | NM_001260485.1 |
|  | Reverse | CACAGGGCAGAAATTGATGACA |  |  |
| *Keap1* | Forward | CGCCTCATCGAGTTCGCTTACAC | 107 | NM_001114671.1 |
|  | Reverse | GCACGGACCACACTGTCAATCTG |  |  |
| *Nrf1* | Forward | CGATGCTTCAGAATTGCCAACTACAG | 125 | XM_021078993.1 |
|  | Reverse | GCGTTGTCTGGATGGTCATCTCAC |  |  |
| *Nrf2* | Forward | CCAATTCAGCCAGCACAACACATC | 149 | XM_003133500 |
|  | Reverse | GACTGAGCCTGGTTAGGAGCAATG |  |  |
| *PC* | Forward | CCGCAAGATGGGAGACAAGGT | 151 | NM_214349.1 |
|  | Reverse | GGAAGCCGTAGGTGTTGGAGAA |  |  |
| *PCK1* | Forward | TCAGCACGACTCCAGCCTTCA | 146 | NM_001123158.1 |
|  | Reverse | GCTCAAGCAGTCTGGGCATTCT |  |  |
| *PCK2* | Forward | ACAGGAGGTTCGTGACATTCGG | 162 | NM_001161753.1 |
|  | Reverse | GTGGTGCTGTGCTCACTTGCTA |  |  |
| *SCD1* | Forward | CTACACAACCACCACTACCATCAC | 152 | NM_009127.4 |
|  | Reverse | GCAAACGCCCAGAGCAAGG |  |  |
| *SOD* | Forward | GTTGGAGACCTGGGCAATGT | 142 | NM_001123124.1 |
|  | Reverse | CGGCCAATGATGGAATGGTC |  |  |
| *TNF-α* | Forward | CCACGCTCTTCTGCCTACTGC | 135 | NM_214022.1 |
|  | Reverse | TCGGCTTTGACATTGGCTACAA |  |  |
| *β-Actin* | Forward | GATCTGGCACCACACCTTCTACAAC | 107 | XM_021086047.1 |
|  | Reverse | TCATCTTCTCACGGTTGGCTTTGG |  |  |

*ACC*, acetyl-CoA carboxylase; *ATGL*, adipose triglyceride lipase; *BESP*, bile salt export pump; *CAT*, catalase; *CYP7A1*, cytochrome P450 family 7 subfamily A member 1; *CYP27A1*, cytochrome P450 family 27 subfamily A member 1; *FAS*, fatty acid synthase; *FXR*, farnesoid X-activated receptor; *GPX*, glutathione peroxidase; *HSL*, hormone-sensitive lipase; *IL-1β,* interleukin 1β; *IL-2,* interleukin 2; *IL-6,* interleukin 6; *IL-10*, interleukin 10; *IFN*-γ, interferon-gama; *Keap1*, kelch-like ECH-associated protein 1; *Nrf1*, nuclear factor erythroid 2-related factor 1; *Nrf2*, nuclear factor erythroid 2-related factor 2; *NTCP*, sodium taurocholate cotransporting polypeptide; *PC*, pyruvate carboxylase; *PCK1*, phosphoenolpyruvate carboxykinase 1; *PCK2*, phosphoenolpyruvate carboxykinase 2; *SCD1*, stearoyl-Coenzyme A desaturase 1; *SOD*, superoxide dismutase; *TNF-α,* tumor necrosis factor-alpha.
